# Microstructure and Thermal Deformation Behavior of Hot-Pressing Sintered Zr-6Al-0.1B Alloy

**DOI:** 10.3390/ma15051816

**Published:** 2022-02-28

**Authors:** Huajun Yan, Wei Wang, Shuangjie Zhang, Shibo Ma, Jianhui Li, Bo Wang

**Affiliations:** 1 Hebei Key Laboratory of Material Near-Net Forming Technology, Hebei University of Science and Technology, Shijiazhuang 050018, China; yanhj22@163.com (H.Y.); lijianhui_97@163.com (J.L.); 2 School of Materials Science and Engineering, Hebei University of Science and Technology, Shijiazhuang 050018, China; wangweicaijia@hebust.edu.cn; 3 Automobile Stamping Die Engineering Research Center, Botou 062150, China

**Keywords:** Zr-6Al-0.1B, hot-pressing sintering, microstructure, thermal deformation behavior

## Abstract

Zr-6Al-0.1B alloy rich in Zr_3_Al phase is prepared by hot-pressing sintering. The thermal deformation behavior of sintered Zr-6Al-0.1B is analyzed by isothermal compression tests at deformation temperatures of 950, 1050, and 1150 °C with strain rates of 0.01, 0.1, and 1 s^−1^. The results indicate that at the early stage of thermal deformation, the stress increases rapidly with the increase of strain and then reaches the peak value. Subsequently, the stress decreases with the increase of strain under the softening effect. On the whole, the true stress-strain curve shifts to the high stress area with the increase of strain rate or the decrease of deformation temperature, so the sintered Zr-6Al-0.1B alloy belongs to the temperature and strain rate sensitive material. For the microstructure evolution of sintered Zr-6Al-0.1B during the isothermal compression, the high strain rate can improve the grain refinement. However, because sintered Zr-6Al-0.1B is a low plastic material, too high strain rate will exceed the deformation capacity of the material, resulting in an increase in defects. The increase of deformation temperature also contributes to grain refinement, but when the temperature is too high, due to the decomposition of Zr_3_Al phase, the deformation coordination of the material decreases, leading to the increase of the probability of the occurrence of defects. This study verified the feasibility of hot-pressing sintering to prepare Zr-6Al-0.1B alloy rich in Zr_3_Al phase and laid the foundation of “hot-pressing sintering + canning hot-extrusion” process of Zr-6Al-0.1B alloy components.

## 1. Introduction

The importance of lightweight, long life, and high reliability to spacecraft components is self-evident. Scholars are working to find more applicative alloy systems with excellent performance in special service environments such as alternating temperature fields, irradiation, and atomic oxygen erosion, so as to maximize the service life of spacecraft components. Currently, materials used by spacecraft components are mostly stainless steel and titanium alloy. However, the feedback of in-orbit use proves that the stainless steel cannot meet the requirements of lightweight, and the titanium alloy has lower hardness and wear resistance, and shorter service life.

Zirconium alloy has better corrosion resistance, creep resistance, and radio resistance, which makes it a potential material for spacecraft components [1,2,3]. Previous studies have revealed that Al in Zr-Al alloy, as a stable element of *α* phase, increases the *α*-*β* phase transition temperature of Zr, and then increases the strength of the alloy [4,5,6]. In addition, Al as a lightweight element, comprehensively optimizes the strength and lightweight properties of the Zr-Al alloy, and the content of Al in the alloy ranges from 1.24 to 2.78 (at.%), Zr-Al alloy can form short-range ordered structure, improve the strength and hardness, but also maintain the particularly important plasticity. Therefore, Zr-Al alloy provides a new direction for the development of spacecraft structural materials [7,8].

The studies of Zr-Al compounds have found that the Zr_2_Al phase has high hardness and almost no plasticity at room temperature. Conversely, the Zr_3_Al phase has a face-centered cubic structure, because of the large number of actuated slip systems and good isotropic properties, so it has excellent plasticity, and is more suitable as an ideal matrix phase for structural materials [9].

Li et al. [10,11] prepared Zr-Al alloy by smelting method, the effects of aluminum content (6.0, 7.0, 8.0 at.%), annealing temperature, and annealing time on the phase composition and mechanical properties of Zr-Al alloy were analyzed. They found that there is no Zr_3_Al phase in the as-casting Zr-Al alloy and almost no plasticity when the phase composition is Zr_2_Al + *α*-Zr. The annealing process is an effective way to obtain the Zr_3_Al + *α*-Zr phases, and the alloy has certain plasticity when the phase composition is Zr_3_Al + *α*-Zr, and with the increase of Al content, the tensile strength and microhardness of Zr_3_Al based alloy increase, while the ductility decreases.

Tewari et al. [12] studied the microstructural evolution of Zr_3_Al upon long-time annealing treatments. The formation of various phases, temperature regime of their stability, chemical composition, and volume fraction of these phases during prolonged annealing were ascertained. The morphology and distribution of the Zr_3_Al phase have been explained on the basis of long-range diffusion as the rate-controlling step, and a pseudobinary phase diagram with varying niobium concentration was developed.

Ren et al. [13] determined the self-diffusion mechanism of Zr_3_Al by systematic first-principles calculations based on density functional theory. The formation energies of four intrinsic point defects were calculated and the relationship between concentration of different point defects and temperature was determined. The effect of defect types on the stability of the system was explored after analyzing the electronic density of different supercells.

Yuan et al. [14] investigated elastic and thermodynamic properties of the L1_2_ type structure Zr_3_Al intermetallic compound under high pressure and temperature using ab initio plane-wave pseudopotential density functional theory (DFT) within the generalized gradient approximation (GGA). They found the elastic modulus and compressional and shear wave velocities are increasing monotonically with increasing pressure.

Arıkan [15] investigated the elastic, electronic, and phonon properties of the intermetallic compounds Zr_3_Al in the L1_2_ structure by employing an ab initio pseudopotential method and a linear-response technique within a generalized gradient approximation (GGA) of the density-functional theory (DFT) scheme. The electronic band structures of Zr_3_Al show that at the Fermi level, a major part of the contribution comes from Zr 4d (Sc 3d) states.

The above studies on Zr-Al alloy show that Zr_3_Al has good comprehensive properties with great application potential. In addition, a large number of studies have shown that multiple alloying can further improve the comprehensive properties of Zr-Al alloy. For example, the addition of Sc element can effectively improve the recrystallization resistance of Zr-Al alloy, and it also can obviously improve the depletion zone of the precipitated phase of Zr-Al binary alloy, making the precipitated phase tend to be evenly distributed. First-principle calculations also confirm that adding Sc in Zr-Al alloy can effectively inhibit the transformation of Al_3_Zr from metastable L1_2_ phase to high temperature stable D0_23_ phase, thus maintaining the recrystallization resistance of the alloy [16,17]. The addition of Ti element can reduce the lattice mismatch, and then stabilize the metastable phase L1_2_-Al_3_Zr [18,19]. Furthermore, the Nb element can improve the ductility when retained by the alloy up to the composition Zr_3_Al_0.5_Nb_0.5_ [20].

In the studies of the effect of adding elements, what appeals to us is the research by Li et al. [21]. They analyzed the effect of B element on the microstructure and mechanical properties of Zr_3_Al-based alloy. The results show that B element can significantly refine the grain size of Zr_3_Al-based alloy. The grain size of Zr_3_Al-based alloy decreases from 500 μm to 30 μm with by adding 0.1 (wt.%) B element. The reason why we are interested is that B element has an excellent grain refining effect, and B element has a lower cost and is more accessible.

At present, the billet of Zr-Al alloy is mainly made by smelting method, which is not only expensive, but also complicated, has low production efficiency and raw material utilization rate. In comparison, hot-pressing sintering is a more flexible preparation method for alloys and composites, with good formability and applicability [22]. Hot-pressing sintering has been successfully applied to the preparation of a variety of alloys and composites, such as, Nickel-based superalloy [23,24], AA6082-ZTA composites [25], Ti-Zr alloys [26], Al-Ti-Zr alloys [27], Ti35Zr28Nb [28], SiC/Al-Zn-Mg-Cu [29], (Ti,Zr)B2-(Zr,Ti)C [30], Zr-1.2Sn1Nb-0.4Fe, and Zr-1.2Bi-1Nb-0.4Fe [31], etc.

In view of the above background, Zr-6Al-0.1B alloy rich in Zr_3_Al phase is prepared by hot-pressing sintering in this study. The microstructure of hot-pressing sintered Zr-6Al-0.1B alloy is analyzed by OM and SEM microscopic imaging techniques. The thermal deformation behavior and microstructure evolution of hot-pressing sintered Zr-6Al-0.1B alloy are analyzed by isothermal compression tests. This study verified the feasibility of hot-pressing sintering to prepare Zr-6Al-0.1B alloy rich in Zr_3_Al phase and laid the foundation of “hot-pressing sintering + canning hot-extrusion” process of Zr-6Al-0.1B alloy components.

## 2. Materials and Methods

### 2.1. Preparation of Zr-6Al-0.1B Alloy

**Figure 1:**
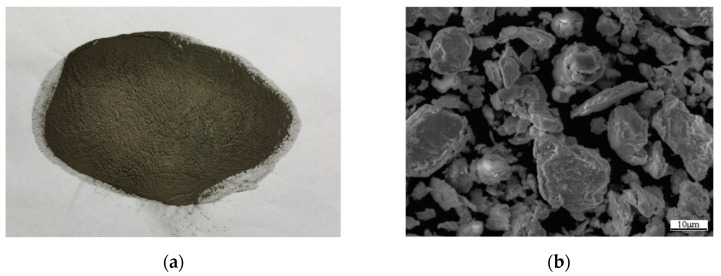
Mixed powder: (**a**) Macroscopic morphology of mixed powder; (**b**) SEM image of mixed powder.

The mixed powder of 93.9% pure zirconium powder (200 mesh) + 6% pure aluminum powder (200 mesh) + 0.1% aluminum boron alloy powder (providing boron element) is obtained through low energy ball milling using a planetary ball mill. The model of planetary ball mill is DECO-30L manufactured by Changsha Deke Instrument Equipment Co., Ltd. in Changsha, Hunan Province, China. First, the three kinds of powder are loaded into a stainless-steel ball mill tank, and several stainless-steel balls are added in ball mill ratio 1:1. Then high purity argon (99.99%) is used to exclude the air in the tank to protect the powder from oxidation and ignition during ball milling [32,33]. The powder mixing time is 30 min at the mixing speed 100 r/min. The macroscopic morphology and SEM image of the mixed powder after ball milling are shown in Figure 1.

Zr-6Al-0.1B alloy is prepared by vacuum hot-pressing sintering using the mixed powder. It should be pointed out that the hardness of Zr_2_Al phase is extremely high and there is almost no plasticity at room temperature. Rather, Zr_3_Al phase is a face-centered cubic structure with more slip systems, so it has good isotropy and more suitable plasticity for processing. Therefore, the key of vacuum hot-pressing sintering is to obtain Zr_3_Al phase, and the sintering temperature is a very crucial process parameter.

**Figure 2:**
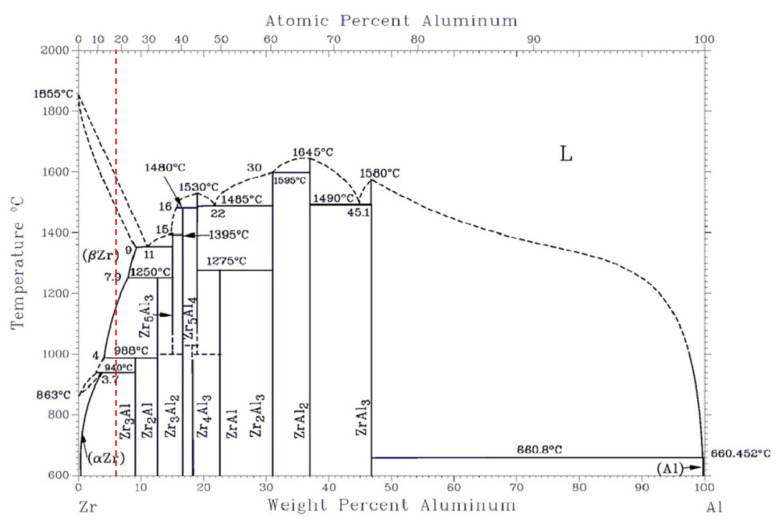
Phase diagram of Zr-Al binary alloy.

The hot-pressing sintering temperature should be ensured within the formation conditions of Zr_3_Al, at the same time, it should be conducive to powder diffusion. According to the phase diagram of Zr-Al binary alloy as shown in Figure 2 [34], Zr has two allotropic crystals, *α*-Zr with HCP crystal structure and *β*-Zr with BCC crystal structure, the phase transition temperature is 863 °C. As a stable element of *α* phase, Al can increase the phase transition temperature. When the mass fraction of Al is 6% (as shown in red dotted line in Figure 2), *α*-Zr transform into*β*-Zr and the peritectic reaction “*β*-Zr + Zr_2_Al⇄Zr_3_Al” occurs when the temperature is higher than 910 °C. However, when the temperature is higher than 1019 °C, the Zr_3_Al is decomposed into *β*-Zr and Zr_2_Al. In Zr-Al alloy, boron element has little effect on phase transition temperature, and its main function is to retard grain growth and prevent oxidization. Therefore, the hot-pressing sintering temperature is selected as 950 °C.

The vacuum hot-pressing sintering equipment is SMVB120/3 manufactured by Zhengzhou Golden Highway Co., Ltd. in Zhengzhou, Henan Province, China. Equipment parameters are as follows, the rated current of equipment is 182 A, the maximum heating power is 120 kW, the maximum sintering temperature is 1200 °C, the maximum compression force is 400 kN, the maximum sintering area (including mold) is 180 cm^3^, the maximum vacuum degree is 76 mmHg.

**Figure 3:**
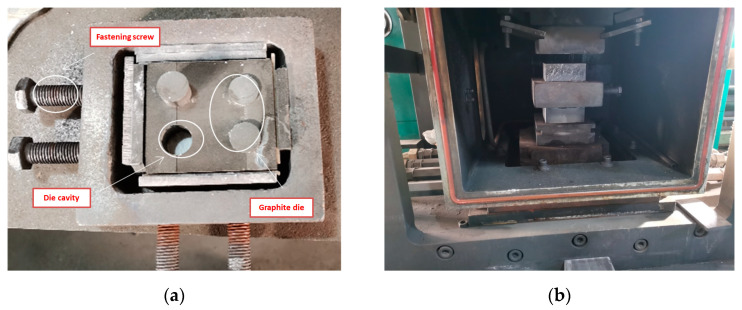
Graphite die and installation: (**a**) Graphite die; (**b**) Die Installation.

Graphite die is used for hot-pressing sintering, as shown in Figure 3. In order to prevent adhesion between metal powder and graphite die during hot-pressing sintering process, the surface of graphite die is evenly coated with a layer of boron nitride, and then mixed powder is loaded into the die, the fastening screw is tightened and put into the vacuum hot-pressing sintering furnace. In order to ensure the sintering effect, the bidirectional pressure mode is used to apply 15 MPa pre-pressure on the graphite die. Then the furnace chamber pumped a vacuum. When the vacuum degree reaches 6 × 10^−^^2^ Pa, the die is heated to 500 °C at a rate of 10 °C/s. Hold for 3 min to eliminate the temperature gradient and pressurize evenly at the same time. When the temperature rises to the specified hot-pressing sintering temperature 950 °C, the pressure rises correspondingly to 40 MPa, and the holding time is 5 min. Stop heating after the holding and pressure process. Stop pressurizing when the temperature drops to 500 °C, and then the furnace body and the graphite die are cooled by the circulation water outside the furnace.

**Figure 4:**
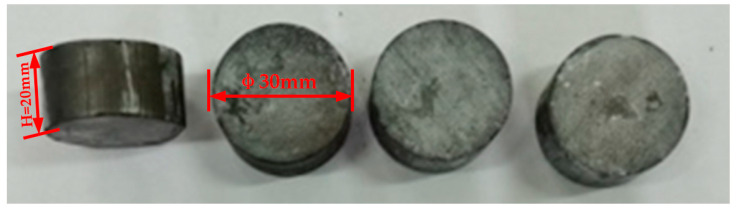
Sintered Zr-6Al-0.1B alloy workpieces.

The sintered Zr-6Al-0.1B alloy workpieces are shown in Figure 4. The workpiece size is Φ30 mm × 20 mm. It can be seen that there is no crack on the surface and the forming effect is good. The theoretical density of Zr-6Al-0.1B is 5.98 g/cm^3^, the actual density of the workpieces is 5.85 g/cm^3^, the compactness reached 97.91%, the hardness is 614.5 HV. The microstructure of the sintered Zr-6Al-0.1B alloy is observed by ZEISS Axio Vert.A1 optical microscope (OM) manufactured by Carl Zeiss in Oberkochen, Germany. And ZEISS ULTRA55 field emission scanning electron microscope (SEM) manufactured by Carl Zeiss in Oberkochen, Germany. The phase analysis is performed by D/MAX-2500 X-ray diffractometer (XRD) manufactured by Rigaku Japan Sales Division in Shibuya-ku, Tokyo, Japan. The distribution of alloying elements is detected by SEM-EDS.

### 2.2. Isothermal Compression Tests

The thermal deformation behavior of sintered Zr-6Al-0.1B alloy is analyzed by isothermal compression tests using a Gleeble-3500 thermal simulation machine manufactured by Data Sciences International, INC. in Delaware, USA. The isothermal compression samples with the size of Φ6 mm × 9 mm are cut by wire cutting from the sintered Zr-6Al-0.1B alloy workpieces. Because Zr-6Al-0.1B is a low plastic alloy, it is necessary to add steel capsule on the outer surface of the sample to prevent the sample from collapsing during isothermal compression, so as to obtain continuous stress-strain data. The addition of steel capsule is also compliance with the process design of “hot-pressing sintering + canning hot-extrusion”.

**Figure 5:**
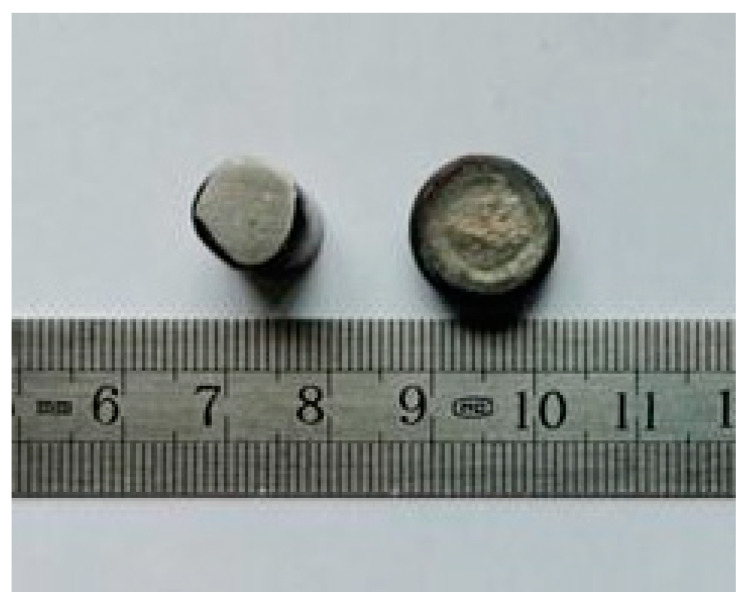
Samples after canning and isothermal compression.

The two end surfaces of the canned samples are covered with graphite flake to eliminate the effect of friction. The thermocouple is welded to the side wall of the samples to control the deformation temperature. The isothermal compression tests are carried out at deformation temperatures of 950, 1050, and 1150 °C with strain rates of 0.01, 0.1, and 1 s^−^^1^. Under vacuum condition, the sample is heated to the deformation temperature at a heating rate of 10 °C/s and held for 30 s to eliminate the temperature gradient. The axial compression ratio is 50%, i.e., the true strain reaches 0.69. After the compression deformation, the specimen is quickly quenched into cold water to keep the microstructure. The samples after canning and isothermal compression are shown in Figure 5.

To observe the microstructures of deformed specimens, they are sectioned parallel to the compression axis using wire cut electrical discharge machining (WEDM) and then the cut surface is polished. The microstructures are observed using ZEISS Axio Vert.A1 optical microscope (OM) and ZEISS ULTRA55 field emission scanning electron microscope (SEM) after etching with metallographic etchant (5% HF + 15% HNO3 + 80% H_2_O).

## 3. Results and Discussion

### 3.1. Microstructure of Sintered Zr-6Al-0.1B Alloy

**Figure 6:**
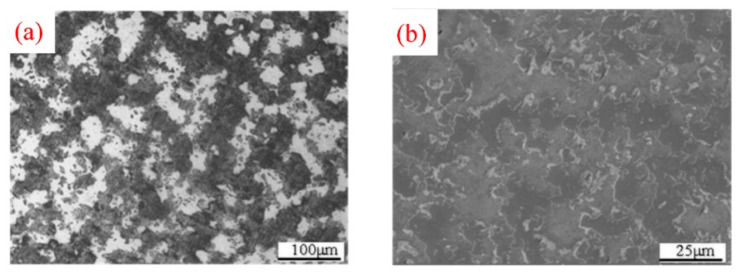
Microstructures of sintered Zr-6Al-0.1B alloy: (**a**) OM image; (**b**) SEM image.

**Figure 7:**
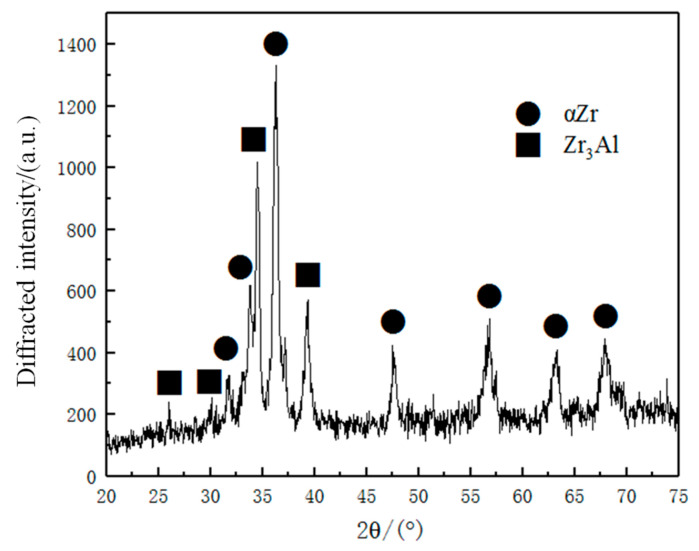
XRD result.

The microstructures of sintered Zr-6Al-0.1B alloy are shown in Figure 6. It can be seen from Figure 6a that the composed of microstructures are the light-colored Zr-Al phase and dark-colored Zr phase, and the distribution is relatively uniform. The morphology of sintered Zr-Al alloy is obviously different from that of melted Zr-Al alloy [9,21]. The XRD result of the workpieces is shown in Figure 7. The results show that the microstructures are mainly composed of Zr_3_Al and *α*-Zr, indicating that under the above hot-pressing sintering conditions, the peritectic reaction “*β*-Zr + Zr_2_Al ⇄Zr_3_Al” is complete, the Zr_2_Al phase is fully transformed into Zr_3_Al phase, and the decomposition of Zr_3_Al phase does not occur.

**Figure 8:**
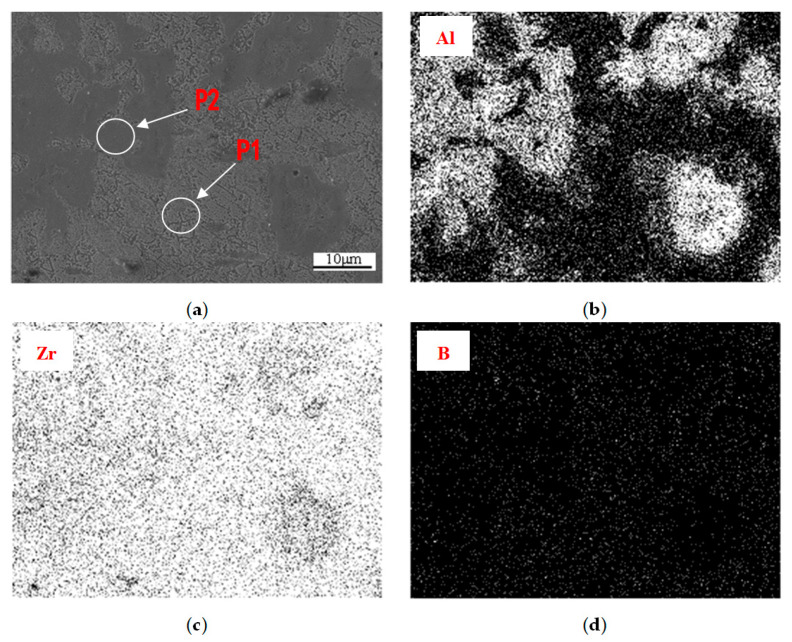
Scanning results of alloying elements: (**a**) Detection region; (**b**) Distribution of Al element; (**c**) Distribution of Zr element; (**d**) Distribution of B element.

EDS analysis is carried out by SEM. The surface scanning results of alloying elements distribution are shown in Figure 8. The atomic percent of elements in regions P1 and P2 are listed in Table 1. The atomic ratios of Zr/Al of the two regions are 16.59 and 3.19, respectively. It can be known that the P1 region is mainly Zr, and the P2 region is mainly Zr-Al. It can be seen from Figure 8b that the distribution of Al element corresponds to the Zr-Al phase distribution, and slight segregation occurs along the grain boundary. As can be seen from Figure 8c, Zr elements are evenly distributed in the form of Zr matrix. As can be seen from Figure 8d, B element is distributed uniformly among alloying elements, but a trace amount of B element is also segregated along the grain boundary of Zr-Al phase, indicating that part of B element is solid dissolved in this phase, which plays a role in reducing the alloy diffusion coefficient and hindering the grain growth of the alloy.

### 3.2. Thermal Deformation Behavior of Sintered Zr-6Al-0.1B Alloy

**Figure 9:**
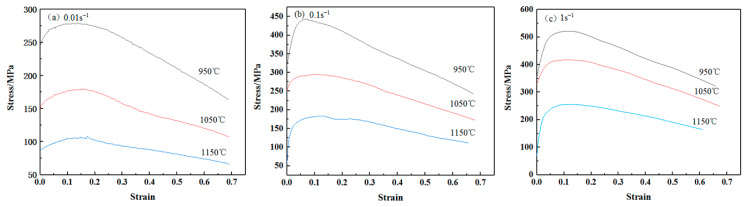
True stress-strain curves of sintered Zr-6Al-0.1B alloy: (**a**) strain rate of 0.01 s^−^^1^; (**b**) strain rate of 0.1 s^−^^1^; (**c**) strain rate of 1 s^−^^1^.

The true stress-strain curves obtained by isothermal compression tests are shown in Figure 9. It can be seen that the variation trend of the true stress-strain curves under different deformation conditions is similar. At the early stage of thermal deformation, the stress increases rapidly with the increase of strain under the influence of work hardening and then reaches the peak value. Subsequently, the stress decreases with the increase of strain under the softening effect of dynamic recovery and dynamic recrystallization. On the whole, the true stress-strain curve shifts to the high stress area with the increase of strain rate or the decrease of deformation temperature, which belongs to the temperature and strain rate sensitive material, that is similar to that of sintered Ti-6Al-4V [35], SiC/AA6061 [36], Al2024-TIN [37], and Fe-25Al-1.5Ta [38], but with a more pronounced softening phenomenon.

It should be pointed out that, due to a steel capsule being used to prevent the failure of the sintered samples during the isothermal compression tests, the obtained stress-strain curves describe the mechanical response of both steel capsule and sintered Zr-6Al-0.1B alloy. It is impossible to separate the contribution of each material to the flow stress. For that reason, the thermal deformation behavior can only be analyzed qualitatively, but not quantitatively. It only provides a reference for the hot-working character of sintered Zr-6Al-0.1B alloy.

### 3.3. Microstructure Evolution of Sintered Zr-6Al-0.1B Alloy

**Figure 10:**
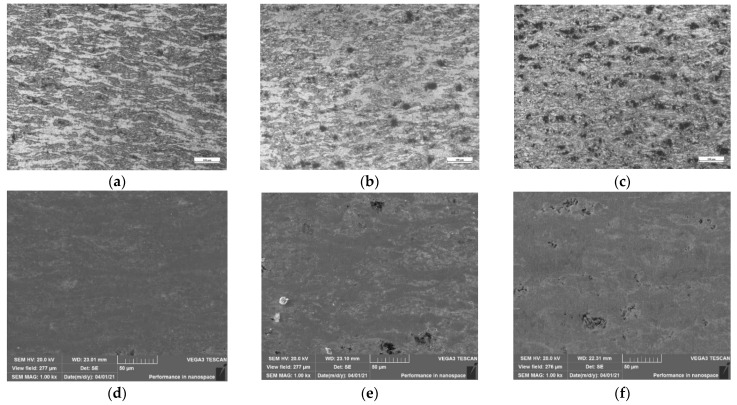
Microstructure of Zr-6Al-0.1B alloy at 1050 °C: (**a**) OM image of 0.01 s^−1^; (**b**) OM image of 0.1 s^−1^; (**c**) OM image of 1 s^−1^; (**d**) SEM image of 0.01 s^−1^; (**e**) SEM image of 0.1 s^−1^; (**f**) SEM image of 1 s^−1^.

The OM and SEM images of isothermal compression samples of Zr-6Al-0.1B alloy at 1050 °C under different strain rates are shown in Figure 10. As shown in Figure 10a,d, when the deformation rate is 0.01 s^−^^1^, most of the grains are unbroken with flat and slender form, and a few unclosed holes are distributed in the matrix. As shown in Figure 10b,e, when the deformation rate is 0.1 s^−1^, the microstructure of the alloy tends to be uniform, internal grain refinement has been improved, but there are obvious holes in the material. As shown in Figure 10c,f, when the deformation rate is 1 s^−1^, the microstructure of the alloy is more uniform and the grain refinement is the highest, but the porosity is obviously increased.

On the whole, for sintered Zr-6Al-0.1B alloy, the high deformation rate can provide a large recrystallization nuclear force, which is conducive to improving the grain refinement. However, because sintered Zr-6Al-0.1B alloy is a low plastic material, a too high deformation rate will exceed the deformation capacity of the material, resulting in an increase in defects of hole. Through an overall consideration, it is suggested to adopt relatively high strain rate in the subsequent selection of plastic processing technology. It is necessary to adopt the processing technology with three compressive stress states, such as extrusion, to improve the degree of material microstructure refinement and inhibit the generation and expansion of holes [39,40,41].

**Figure 11:**
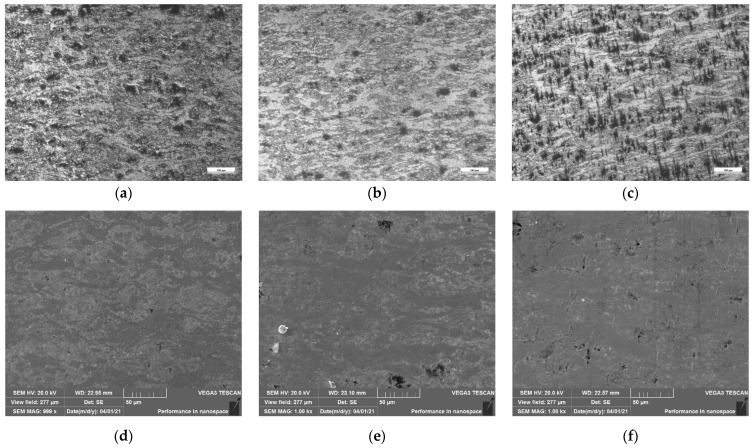
Microstructure of Zr-6Al-0.1B alloy at 0.1 s^−1^: (**a**) OM image of 950 °C; (**b**) OM image of 1050 °C; (**c**) OM image of 1150 °C; (**d**) SEM image of 950 °C; (**e**) SEM image of 1050 °C; (**f**) SEM image of 1150 °C.

The OM and SEM images of isothermal compression samples of Zr-6Al-0.1B alloy at 0.1 s^−1^ under different deformation temperatures are shown in Figure 11. As shown in Figure 11a,d, when the deformation temperature is 950 °C, the deformation of the material is more uniform, the Zr-Al phase can be evenly distributed, and the internal holes are relatively few. As shown in Figure 11b,e, when the deformation temperature is 1050 °C, although the increase of deformation temperature promotes the migration of grain boundary and accelerates the dynamic recrystallization inside the material, the grain refinement degree is slightly improved. However, Zr and bright white Zr-Al phases are fibrous and have poor microstructure uniformity. As shown in Figure 11c,f, when the deformation temperature is 1150 °C, dynamic recrystallization is more sufficient and grain size is more refined. However, a large number of obvious holes appear in the material, which is because at this temperature, Zr_3_Al phase with good plasticity is decomposed into Zr_2_Al phase with poor plasticity. There is a great difference between the plasticity of Zr_2_Al phase and Zr phase, so the deformation coordination of the material is reduced, leading to the generation of defects.

On the whole, the increase of deformation temperature contributes to grain refinement. However, when the temperature is too high, due to the decomposition of Zr_3_Al phase the deformation coordination of the material decreases. It will lead to the increase of the probability of the occurrence of defects, such as holes. Through an overall consideration, it is suggested that the deformation temperature should be higher than the phase transition temperature of *α*-Zr to *β*-Zr (863 °C), but lower than the decomposition temperature of Zr_3_Al (1019 °C). *β*-Zr and Zr_3_Al both have relatively good plasticity, which is beneficial to improve the uniformity of microstructure and reduce the possibility of defects [9,10,11].

## 4. Conclusions

(1) Zr-6Al-0.1B alloy rich in Zr_3_Al phase can be prepared by vacuum hot-pressing sintering using the following parameters: powder composition is 93.9% pure zirconium powder (200 mesh) + 6% pure aluminum powder (200 mesh) + 0.1% aluminum boron alloy; powder mixing time is 30 min with ball mill ratio 1:1 at the mixing speed 100 r/min; the hot-pressing sintering temperature is 950 °C with sintering pressure 40 MPa and sintering time 5 min. The density of the sintered Zr-6Al-0.1B alloy is 5.85 g/cm^3^, the compactness can reach 97.91%, the hardness can reach 614.5 HV.

(2) The sintered Zr-6Al-0.1B alloy belongs to the temperature and strain rate sensitive material. At the early stage of thermal deformation, the stress increases rapidly with the increase of strain under the influence of work hardening and then reaches the peak value. Under the softening effect of dynamic recovery and dynamic recrystallization, the stress decreases with the increase of strain. On the whole, the true stress–strain curve shifts to the high stress area with the increase of strain rate or the decrease of deformation temperature.

(3) The high deformation rate is conducive to improving the grain refinement of sintered Zr-6Al-0.1B alloy, but a too high deformation rate will exceed the deformation capacity of the material, resulting in an increase in defects. The increase of deformation temperature contributes to grain refinement, but when the temperature is too high, due to the decomposition of Zr_3_Al phase, the deformation coordination of the material decreases, leading to the increase of the probability of the occurrence of defects.

## Figures and Tables

**Table 1:** Atomic ratios of elements in regions P1 and P2 (at.%).

Region	Zr	Al	B
P1	93.42	5.63	0.95
P2	75.66	23.72	0.62

## Data Availability

The data presented in this study are available on request from the corresponding author.
